# Evolution of Artistic and Athletic Propensities: Testing of Intersexual Selection and Intrasexual Competition

**DOI:** 10.3389/fpsyg.2022.925862

**Published:** 2022-07-07

**Authors:** Marco Antonio Correa Varella, Zuzana Štěrbová, Klára Bártová, Maryanne L. Fisher, Jaroslava Varella Valentova

**Affiliations:** ^1^Department of Experimental Psychology, Institute of Psychology, University of São Paulo, São Paulo, Brazil; ^2^Department of Psychology, Faculty of Arts, Charles University, Prague, Czechia; ^3^Department of Zoology, Faculty of Science, Charles University, Prague, Czechia; ^4^Department of Psychology and Life Sciences, Faculty of Humanities, Charles University, Prague, Czechia; ^5^Department of Psychology, Saint Mary’s University, Halifax, NS, Canada

**Keywords:** arts, aesthetic behavior, sexual selection, aggression, mating, creativity, sport, ornament

## Abstract

Since Darwin proposed that human musicality evolved through sexual selection, empirical evidence has supported intersexual selection as one of the adaptive functions of artistic propensities. However, intrasexual competition has been overlooked. We tested their relative importance by investigating the relationship between the self-perceived talent/expertise in 16 artistic and 2 sports modalities and proxies of intersexual selection (i.e., mate value, mating and parenting efforts, sociosexuality, and number of sexual partners) and intrasexual competition (i.e., aggressiveness, intrasexual competitiveness) in heterosexuals. Participants were 82 Brazilian men, 166 Brazilian women, 146 Czech men, and 458 Czech women (Mage = 26.48, SD = 7.12). Factor analysis revealed five factors: Literary-arts (creative writing, humor, acting/theater/film, poetry, storytelling), Visual-arts (painting/drawing, sculpting, handcrafting, culinary arts, architecture design), Musical-arts (playing/instruments, singing, dance, whistling), Circus-arts (juggling, acrobatics), and Sports (individual, collective). Multivariate General Linear Model (GLM) showed more associations of the arts to intersexual selection in women and to intrasexual selection in men, and overall more relationships in women than in men. In women, literary and musical-arts were related to elevated inter- and intrasexual selections proxies, visual and circus-arts were related to elevated intersexual selection proxies, and sports were related to intrasexual selection proxies. In men, literary-arts and sports were related to elevated inter- and intrasexual selection proxies, musical-arts were related to intrasexual proxies, and circus-arts were related to intersexual proxies; visual-arts did not have predictors. Although present in both sexes, each sexual selection component has different relative importance in each sex. Artisticality functions to attract and maintain long/short-term partners, and to compete with mating rivals.

## Introduction

Recently, an increasing number of theoretical propositions and empirical studies have developed to explain the evolved aspects of artistic propensities. Some researchers support the hypothesis that artistic activities result from non-artistic pre-existing psychological capacities recently co-opted as a non-specialized by-product to generate human artistic output (e.g., Pinker, 2004; Souza, 2004; Hodgson and Verpooten, 2015). Others argue for the existence of a set of ancestral psychological capacities that are specialized in processing artistic information (e.g., Eibl-Eibesfeldt, 1989; Zahavi and Zahavi, 1999; Martindale et al., 2007; Dutton, 2009; Davies, 2012; Sütterlin et al., 2014; Hogh-Olesen, 2018; Richards, 2019; Varella, 2021). The main proposed ancestral adaptive values are related to survival benefits (e.g., in-group cohesion, parental bonding, cognition and health improvement) and to reproductive advantages (e.g., mate selection, intrasexual competition) (Dissanayake, 2008; Varella et al., 2010, 2011, 2017; Menninghaus, 2019; De Tiège et al., 2021; Kalinowski et al., 2021). The different evolutionary perspectives are not mutually exclusive (Menninghaus, 2019), so dismissing one perspective does not necessarily support another (Varella et al., 2017). Not every behavioral tendency has adaptive value, not every ancestral adaptation is currently adaptive, and ancestral adaptive value does not need to be consciously represented in one’s mind for one to behave adaptively (Varella et al., 2012, 2013). Only corroborative and cumulative empirical evidence can strengthen the case for exaptationist and adaptationist propositions (cf. Andrews et al., 2002; Schmitt and Pilcher, 2004).

Roughly 150 years ago, Darwin (1871) proposed that animal, including human, body and psychobehavioral ornaments evolved mostly through sexual selection by female mate choice. He integrated various sources of evidence and argued that the pleasures, the universality, the prehistoric antiquity, the emotions evoked, and the adaptive convergence of musical and dancing behaviors “become intelligible to a certain extent, if we may assume that musical tones and rhythm were used by our half-human ancestors, during the season of courtship, when animals of all kinds are excited not only by love, but by the strong passions of jealousy, rivalry, and triumph” (p. 572). By stressing jealousy, rivalry, and triumph alongside love, Darwin suggested a plurality of evolutionary mechanisms that includes both inter and intrasexual selection.

### Intersexual Selection

Artistic propensities could have evolved as an ornament. Converging evidence has mostly corroborated intersexual selection as one of the possible processes influencing the evolution of human aesthetic creativity and artistic propensities (Low, 1979; Karamihalev, 2013). The mere listening of enjoyable background music leads to: increased testosterone levels in women (Fukui, 2001), women’s increased attractiveness ratings of male photographs (May and Hamilton, 1980; Marin et al., 2017), and women’s increased desire to go on a romantic date with the individuals in the photographs; no such effects were found in men (Marin et al., 2017). Groovy background music increases women’s desire to meet again a speed dating partner, and the synchronization of their body sway predicts their interest in a long-term relationship beyond perceived attractiveness (Chang et al., 2021). Even seeing a guitar impacts on women’s mating decisions: women tend to reply positively to a friendship request on social network when the profile picture shows a man holding a guitar (Tifferet et al., 2012). Further, activated mating motivations increase creative displays in men and women; both short-term and long-term contexts increase men’s creativity, but only a high-quality mate and a long-term context increase creativity in women (Griskevicius et al., 2006).

Regarding mate preferences, “artistic-intelligent” is the third most preferred factor among married couples and explains 4.5% of the variance behind “kind and considerate” (16%) and “socially exciting” (6.9%). In an unmarried sample, “creative” is ranked the 7th most important mate preference trait (Buss and Barnes, 1986). Across 37 cultures, men and women rank “artistic” and “creative” as the 6th and 7th most important characteristics in a romantic partner, respectively (Buss et al., 1990). In a 25-year follow up study in India, the preferences for “creative and artistic” increased in both sexes (Kamble et al., 2014), and 30 years later in Brazil, the preference for “creative and artistic” increased in men and decreased in women (Souza et al., 2016). Both sexes prefer more ornamental and aesthetic types of creativity (e.g., writing music, poetry, drawing) in a prospective sexual partner than “applied/technological” and “everyday/domestic” forms of creativity; although women prefer relatively more the first type, while men prefer more the third type of creativity (Kaufman et al., 2016). Women’s preferences are even influenced by their fertility status: those in their maximally fertile phase prefer “creativity” over “wealth” for a short-term relationship, but not for a long-term one (Haselton and Miller, 2006), and they prefer composers of complex musical pieces for short-term relationships (Charlton, 2014). Further, women in their peak fertility exhibit increased creativity (Galasinska and Szymkow, 2021) and more attractive dance movements (Miller et al., 2007; Fink et al., 2012).

Importantly, after satisfying mate preference necessities (e.g., intelligence, income, attractiveness), men and women prefer creativity in a romantic partner (Li et al., 2002; Thomas et al., 2020). Men’s creativity increases women’s rating of attractiveness and mate appeal (Prokosch et al., 2009; Watkins, 2017), independently of intelligence (Prokosch et al., 2009), and even compensates for low facial attractiveness (Watkins, 2017). The reverse also may be supported, as men also perceived creative women as more attractive (Watkins, 2017). Women appreciate funny men, while men value women who appreciate their jokes (Bressler and Balshine, 2006; Bressler et al., 2006; Hone et al., 2015). Men and women prefer mates who privately sing or play musical instruments (Bongard et al., 2019). Individuals who like to sing more, who sing with higher pitch modulation, and higher-pitched singing in women are perceived as more attractive (Valentova et al., 2019). In men, physical size positively predicts speech and singing attractiveness (Valentova et al., 2019). Last, for dance, good male dancers receive more attention and are rated by women as more attractive and masculine than bad dancers (Weege et al., 2012). Dancing women who are rated as more attractive and feminine receive more visual attention (Röder et al., 2016). Dance movements of physically stronger men are evaluated as more attractive by women (Hugill et al., 2009; Weege et al., 2015).

Regarding the connection of creativity and artistic propensities with sexual strategies and mating outcomes, men with higher-pitched singing have higher sociosexuality (i.e., variety of casual sex) (Valentova et al., 2019). Men and women with greater humor production have more short-term uncommitted sex, and the ability to produce humor in both sexes mediates the positive effects of intelligence and mating success (Greengross and Miller, 2011). Poets and artists of both sexes tend to have more sexual partners than control groups, and higher levels of creative engagement correlate with higher numbers of sexual partners (Nettle and Clegg, 2006). Men exhibiting a stronger tendency to engage in everyday forms of creative activity tend to report more sexual partners in the last year, but no corresponding result is found for women (Beaussart et al., 2012). Successful male visual artists have more sexual partners and have a more long-term oriented sexual strategy than less successful visual artists, with no effect found in female visual artists (Clegg et al., 2011). High music achievement in men relates to higher number of children, and, in both sexes, music aptitude and achievement relate to a long-term mating orientation, i.e., low number of sex-partners, older age of first intercourse, and restricted sociosexuality (Mosing et al., 2015).

Despite the inter-sexual selection hypothesis being well corroborated empirically, some predictions have not been supported. These conflicting findings indicate the necessity for further research. For instance, visually creative individuals in a non-industrialized semi-nomadic Kenyan tribe with natural fertility tend to have fewer children (Lebuda et al., 2021). Further, individuals with greater musical ability have lower mating success (Mosing et al., 2015). Others report no effect of women’s conception risk on their short-term preferences for male creativity (Prokosch et al., 2009). Last, the attractiveness of one’s singing voice is not related to their sexual strategies (Valentova et al., 2019), and musicians and non-musicians do not differ in the number of sex partners (Harrison and Hughes, 2017).

### Intrasexual Competition

The maintenance of long-term relationships, parental effort, and intrasexual competition are central, although understudied, domains of sexual selection (Andersson, 1994; Puts, 2010; Varella et al., 2017). Artistic propensities could have evolved as an armament in the prestige competition (De Block and Dewitte, 2007; Varella et al., 2017; Winegard et al., 2018). Both sexes should compete intrasexually using the traits that the other sex finds desirable, and “artistic” and “creative” are among the desired traits in a mate (Buss and Barnes, 1986; Buss et al., 1990). Indeed, the same women’s vocal parameters that men find attractive (i.e., femininity) are used by other women to track the threat of potential rivals (Puts et al., 2011). Self-promotion *via* aesthetics and beauty can be both an intersexual ornament and an intrasexual armament in both sexes, although mostly in women (Varella et al., 2017; Mafra et al., 2020). Fisher and Candea (2012) showed that popular women musicians include in their song lyrics topics of intrasexual competition such as mate manipulation, self-promotion, and competitor derogation/manipulation. Nevertheless, little effort has been made to empirically explore intrasexual selection hypotheses, and to contrast the effects of inter and intrasexual components.

### Comparing Intersexual and Intrasexual Selection

Chen and Chang (2015) found that, in men, creativity (i.e., general originality) increases in an intersexual vs. intrasexual situational condition, but in women, creativity is the same in both situations. Moreover, creative males exhibit a mating strategy bias toward intersexual courtship compared to intrasexual competition. Even beyond the arts, there has been minimal work that compares the two components of sexual selection. Sports, like the arts, can be seen as play/leisure and profession, dependent on specific motivation, talent, skill, and as sexually selected cultural displays and honest fitness indicators (Zahavi and Zahavi, 1999; Miller, 2000; De Block and Dewitte, 2007; Lombardo, 2012; Hsu and Valentova, 2020; Moraes et al., 2021; Hsu et al., 2022). There also have been theoretical propositions suggesting that both intersexual and intrasexual selection might have influenced the evolution of athletic propensities (e.g., Lombardo, 2012; Apostolou, 2015), however, no direct empirical tests have been conducted beyond sex differences (e.g., Apostolou, 2015).

The few studies comparing the strength of inter- and intrasexual selection were performed for sexually dimorphic male-biased traits. Concerning bodily traits, there is a higher relevance of intrasexual competition than intersexual selection; physical dominance leads to mating success (Kordsmeyer et al., 2018). For the evolution of human voice characteristic, although the low fundamental frequency predicts attractiveness and dominance ratings, the intrasexual component *via* physical dominance exhibit a stronger role than mate selection (Puts, 2010; Hodges-Simeon et al., 2011; Puts et al., 2016). Therefore, the relative importance of inter- and intrasexual selection in the case of artistic and athletic propensities requires empirical investigation.

### Aims

We investigated associations of proxies of intersexual selection and intrasexual competition with individual variation in artistic and athletic tendencies in a cross-cultural sample (Brazil and the Czechia). Based on the available evidence, we hypothesized that in both sexes artistic and athletic propensities would be more associated with traits related to intersexual selection (e.g., mate value, number of partners) than intrasexual selection (e.g., competitiveness, aggressiveness).

## Method

### Participants

Participants were 82 Brazilian men (Mage = 28.35, SD = 5.91), 166 Brazilian women (Mage = 26.05, SD = 6.43), 146 Czech men (Mage = 26.74, SD = 7.45), and 458 Czech women (Mage = 26.52, SD = 7.60), all of whom identified as heterosexuals (18–69 years old). Unfinished participation (*n* = 472), and non-heterosexual participants (*n* = 104) were excluded from this study. Intrasexual and intersexual selections work in different ways in non-heterosexual individuals (cf. Semenyna et al., 2020).

### Procedures

Participants were recruited between April 2013 and April 2014 through social media. Upon informed consent, they voluntarily answered the anonymous online questionnaires in Qualtrics (Provo, UT). This study was a part of a bigger project (cf. Valentova et al., 2020), thus only information relevant to the present study is provided. The procedure took about 50 min; there was no payment for participation. The procedure was the same in both countries.

### Materials

All questionnaires were back-translated and adjusted to Brazilian-Portuguese and Czech language. Participants provided basic socio-demographic data: age, sex, and relationship status.

Inspired by the Creative Achievement Questionnaire (Carson et al., 2005), we asked participants to indicate their self-perceived level of talent/experience in 18 artistic leisure activities (i.e., 16 artistic and 2 sports modalities) using a scale from 0 (no talent/experience) to 10 (very much talent/experience). Principal component analysis with Varimax rotation grouped the leisure activities according to eigenvalues > 0.1 (see Table 1). The solution yielded 5 factors named as follows: (PC1) Literary-arts (creative writing, humor, acting in theater/film, poetry, storytelling), (PC2) Visual-arts (painting/drawing, sculpting, handcrafting, culinary arts, architecture design), (PC3) Musical-arts (playing instruments, singing, dance, whistling), (PC4) Sports (individual and collective sports), (PC5) Circus-arts (juggling, doing acrobatics).

**TABLE 1:** Factor loadings of the 18 leisure activities after principal component analysis.

	Component loadings					
	PC1	PC2	PC3	PC4	PC5	Uniqueness
Creative writing	0.804					0.320
Storytelling	0.732					0.398
Humor	0.679					0.412
Poetry	0.650					0.439
Acting theater film	0.512					0.620
Handcrafting		0.756				0.391
Painting and drawing		0.712				0.437
Architectural design		0.702				0.457
Sculpting		0.666				0.341
Culinary arts		0.550				0.566
Singing			0.848			0.221
Musical instrument playing			0.767			0.322
Dance			0.502			0.528
Whistling			0.459			0.682
Individual sport				0.752		0.364
Collective sport				0.751		0.379
Juggling					0.768	0.319
Acrobatics					0.705	0.340

*Applied rotation method is varimax.*

The Self-Perceived Mate Value questionnaire was inspired by previous measures testing this concept (see Fernandez et al., 2014). The participants reported how easy it would be (1 = *extremely difficult*; 7 = *extremely easy*) to find a short-term (i.e., “If you were single, how easy would it be for you to find a short-term mate for romance?” and “If you were single, how easy would it be for you to find a short-term mate for only sex?”) and a long-term mate (i.e., “If you were single, how easy would it be for you to find a potential long-term mate?” and “If you were single, how easy would it be for you to find a long-term relationship potentially leading to marriage?”). Using the same scale, they also answered a more general question “How easy would it be to find a potential partner at this moment, in the city where you live if you were single.” Finally, the participants self-rated their physical attractiveness on a scale from 1 (*Not at all attractive*) to 7 (*Very attractive*). Factor analysis with varimax rotation yielded a 2-factor solution with eigenvalues > 1. (3.008; 1.220) accounting for 70.465% of the total variance. The factors were named Short-term mate value and Long-term mate-value and their regression scores entered into further analyses.

The Sociosexual Orientation Inventory-Revised (SOI-R; Penke and Asendorpf, 2008) measures the propensity toward uncommitted sexual variety using 9 items completed *via* 9-point scales. The SOI-R is divided into sociosexual behavior (α = 0.790), attitudes (α = 0.777), and desires (α = 0.828).

The Brief Life-History Scale (Kruger, 2017) has 4 items for each dimension: mating effort (α = 0.687; e.g., “sleep with a large number of people in your lifetime”) and parenting effort (α = 0.574; e.g., “caring and emotionally supportive in a long-term relationship”), measured on a 7-point scale (1 = *not at all*; 7 = *very much*) for how each item describe the participants.

The Intrasexual Competition Scale (Buunk and Fisher, 2009) has 12 sex-specific items (e.g., “I want to be just a little better than other men/women.”) measuring intrasexual competitiveness on a 7-point scale (1 = *not at all applicable*; 7 = *completely applicable*). Factor analysis with varimax rotation yielded a 3-factor solution with eigenvalues > 1. (4.862; 1.474; 1.061) accounting for 61.644% of the total variance. The factors were named as Attractiveness envy, Superiority, and Status protection. Their regression scores entered into further analyses.

The Brazilian adaptation of the Buss-Perry Aggression Questionnaire (Gouveia et al., 2008) has 26 items answered on 5-point scales (1 = *extremely uncharacteristic of me*; 5 = *extremely characteristic of me*) loading onto 4 subscales: anger (α = 0.834; e.g., “Some of my friends think I’m a hothead”), hostility (α = 0.688; e.g., “I am suspicious of overly friendly strangers”), physical aggression (α = 0.813; e.g., “If somebody hits me, I hit back”), and verbal aggression (α = 0.603; e.g., “I often find myself disagreeing with people”). The Czech translation kept the same items as the Brazilian version. The average of the items per subscale entered further analyses.

### Analyses

Analyses were run using SPSS 26.0 (IBM Corp., Armonk, NY, United States). To test for a potential effect of sex, we performed a multivariate General Linear Model (GLM) with the 5 leisure activities factors (viz., literary, musical, visual, and circus arts, and sports) as dependent variables, sex as a factor, and age as a covariate.

To test the influence of indicators of inter- and intrasexual selection on artistic talent/experience factors, we ran a multivariate GLM for each sex separately with artistic talent/experience factors as dependent variables, while two mate value subscales (short-term and long-term), life history sub-scales (mating and parenting efforts), sociosexuality (behavior, desires and attitudes), numbers of long-term and short-term partners, four aggressiveness subscales (anger, hostility, physical, and verbal aggression), three intrasexual competitiveness subscales (attractiveness envy, superiority, and status protection), and age entered as predictors. We report partial Eta-squared (ηp2) and Observed power as effect size estimators.

## Results

### Influence of Sex and Age on Leisure Activities

We created a multivariate GLM with the five leisure activities factors as dependent variables, sex as factor, and age as a covariate. Age had significant negative effect on Musical arts (*F* = 10.15, *df* = 1, *p* = 0.001, η_p_^2^ = 0.012), and Circus arts (*F* = 5.26, *df* = 1, *p* = 0.022, η_p_^2^ = 0.006), and no significant effect on Literary arts (*F* = 3.18, *df* = 1, *p* = 0.075, η_p_^2^ = 0.004), Visual arts (*F* = 3.55, *df* = 1, *p* = 0.060, η_p_^2^ = 0.004), or Sports (*F* = 1.78, *df* = 1, *p* = 0.183, η_p_^2^ = 0.002). Sex had a significant effect on all the leisure activities, namely, Literary arts (*F* = 24.92, *df* = 1, *p* < 0.001, η_p_^2^ = 0.030), Visual arts (*F* = 52.34, *df* = 1, *p* < 0.001, η_p_^2^ = 0.060), Musical arts (*F* = 17.05, *df* = 1, *p* < 0.001, η_p_^2^ = 0.020), Sport (*F* = 7.29, *df* = 1, *p* = 0.007, η_p_^2^ = 0.009), and Circus arts (*F* = 68.43, *df* = 1, *p* < 0.001, η_p_^2^ = 0.077). Specifically, women showed higher score in Visual arts and Musical arts, while men scored higher in Literary arts, Sport, and Circus arts. Due to these differences, we performed analyses separately for men and women, controlling for age.

### Influence of Intrasexual Competition and Intersexual Selection Indicators on Leisure Activities

We constructed two multivariate GLMs separately for men and women, with the five leisure activities as dependent variables, and indicators of intersexual selection and intrasexual competition as predictors.

In women (*N* = 541), the model (Pillai’s Trace = 0.039, *F* = 4,213, *p* = 0.001, η_p_^2^ = 0.039, Observed power = 0.960) showed that Literary arts were predicted positively by Short-term mate value, SOI-R Desire, IC-Superiority, and negatively by SOI-R Attitude. Visual arts were predicted positively by parenting effort, and negatively by anger and IC-Attractiveness envy. Musical arts were predicted positively by Short-term mate value and IC-Attractiveness envy, and negatively by hostility and SOI-R Attitude. Circus arts were predicted positively by the number of long-term partners, and negatively by Parenting effort and SOI-R Attitude. Sport was predicted positively by verbal aggression, and negatively by anger. See Table 2.

**TABLE 2:** Parameter estimates of the multivariate general linear model (GLM) model indicating the effect of the independent variables on artisticality in women.

Dependent variable	Parameter	B	SE	*t*	*p*	95% CI		Partial eta^2^	Observed power
						Lower bound	Upper bound		
Literary arts	Intercept	–0.866	0.394	–2.299	0.028	–1.640	–0.092	0.009	0.593
	Age	0.022	0.006	3.445	0.001	0.010	0.035	0.022	0.930
	LH parenting effort	0.028	0.044	0.631	0.528	–0.059	0.115	0.001	0.097
	LH mating effort	0.060	0.048	1.249	0.212	–0.034	0.154	0.003	0.239
	SOI-R behavior	–0.007	0.034	–0.217	0.828	–0.073	0.059	0.000	0.055
	SOI-R attitude	–0.054	0.023	–2.309	0.021	–0.100	–0.008	0.010	0.635
	SOI-R desire	0.074	0.029	2.570	0.010	0.017	0.130	0.012	0.728
	N of long-term relationships	–0.075	0.043	–1.740	0.082	–0.159	0.010	0.006	0.412
	N of short-term relationships	0.009	0.007	1.387	0.166	–0.004	0.022	0.004	0.283
	Mate value–Long term	0.090	0.046	1.957	0.051	0.000	0.180	0.007	0.497
	Mate value–Short term	0.194	0.050	3.883	0.000	0.096	0.292	0.028	0.972
	BPA anger	–0.095	0.061	–1.568	0.118	–0.214	0.024	0.005	0.347
	BPA physical aggression	0.050	0.078	0.645	0.519	–0.103	0.203	0.001	0.099
	BPA verbal aggression	0.128	0.069	1.850	0.065	–0.008	0.264	0.006	0.455
	BPA hostility	–0.063	0.066	–0.959	0.338	–0.191	0.066	0.002	0.160
	IC attractiveness envy	–0.027	0.047	–0.571	0.568	–0.118	0.065	0.001	0.088
	IC superiority	0.150	0.045	3.358	0.001	0.062	0.238	0.021	0.918
	IC status protection	–0.065	0.043	–1.509	0.132	–0.150	0.020	0.004	0.325
Visual arts	Intercept	–0.603	0.413	–1.461	0.145	–1.414	0.208	0.004	0.308
	Age	–0.001	0.007	–0.163	0.871	–0.014	0.012	0.000	0.053
	LH parenting effort	0.130	0.046	2.807	0.005	0.039	0.221	0.015	0.800
	LH mating effort	0.070	0.050	1.397	0.163	–0.029	0.169	0.004	0.286
	SOI-R behavior	–0.035	0.035	–1.008	0.314	–0.105	0.034	0.002	0.172
	SOI-R attitude	–0.015	0.025	–0.618	0.537	–0.063	0.033	0.001	0.095
	SOI-R desire	0.002	0.030	0.065	0.949	–0.057	0.061	0.000	0.050
	N of long-term relationships	0.042	0.045	0.941	0.347	–0.046	0.131	0.002	0.156
	N of short-term relationships	–0.010	0.007	–1.379	0.169	–0.023	0.004	0.004	0.280
	Mate value–Long term	0.034	0.048	0.711	0.477	–0.060	0.129	0.001	0.109
	Mate value–Short term	0.060	0.052	1.142	0.254	–0.043	0.163	0.002	0.207
	BPA anger	–0.150	0.064	–2.356	0.019	–0.275	–0.025	0.011	0.652
	BPA physical aggression	0.093	0.082	1.145	0.253	–0.067	0.254	0.003	0.208
	BPA Verbal aggression	0.091	0.073	1.254	0.210	–0.052	0.234	0.003	0.240
	BPA hostility	0.025	0.069	0.357	0.721	–0.110	0.159	0.000	0.065
	IC attractiveness envy	–0.096	0.049	–1.965	0.050	–0.192	–1.517E-5	0.007	0.501
	IC superiority	0.092	0.047	1.953	0.051	–0.001	0.184	0.007	0.496
	IC status protection	0.055	0.045	1.213	0.226	–0.034	0.143	0.003	0.228
Musical arts	Intercept	1.042	0.408	2.554	0.011	0.241	1.844	0.012	0.722
	Age	–0.016	0.007	–2.400	0.017	–0.029	–0.003	0.011	0.669
	LH parenting effort	0.025	0.046	0.549	0.584	–0.065	0.115	0.001	0.085
	LH mating effort	0.060	0.050	1.204	0.229	–0.038	0.158	0.003	0.225
	SOI-R behavior	–0.025	0.035	–0.728	0.467	–0.094	0.043	0.001	0.112
	SOI-R attitude	–0.075	0.024	–3.073	0.002	–0.122	–0.027	0.018	0.866
	SOI-R desire	0.013	0.030	0.425	0.671	–0.046	0.071	0.000	0.071
	N of long-term relationships	0.004	0.044	0.095	0.924	–0.083	0.092	0.000	0.051
	N of short-term relationships	–0.008	0.007	–1.223	0.222	–0.022	0.005	0.003	0.231
	Mate value–Long term	0.019	0.048	0.394	0.694	–0.075	0.112	0.000	0.068
	Mate value–Short term	0.119	0.052	2.305	0.022	0.018	0.221	0.010	0.634
	BPA anger	0.002	0.063	0.039	0.969	–0.121	0.126	0.000	0.050
	BPA physical aggression	0.044	0.081	0.541	0.588	–0.115	0.202	0.001	0.084
	BPA verbal aggression	–0.020	0.072	–0.281	0.779	–0.161	0.121	0.000	0.059
	BPA hostility	–0.154	0.068	–2.264	0.024	–0.287	–0.020	0.010	0.618
	IC attractiveness envy	0.116	0.048	2.410	0.016	0.021	0.211	0.011	0.672
	IC superiority	0.025	0.046	0.547	0.584	–0.066	0.116	0.001	0.085
	IC status protection	0.000	0.045	–0.006	0.995	–0.088	0.087	0.000	0.050
Sports	Intercept	–1.067	0.399	–2.671	0.008	–1.852	–0.282	0.013	0.760
	Age	0.002	0.007	0.268	0.789	–0.011	0.015	0.000	0.058
	LH parenting effort	0.080	0.045	1.795	0.073	–0.008	0.168	0.006	0.433
	LH mating effort	0.028	0.049	0.567	0.571	–0.068	0.123	0.001	0.087
	SOI-R behavior	0.023	0.034	0.664	0.507	–0.044	0.089	0.001	0.102
	SOI-R attitude	0.000	0.024	0.018	0.986	–0.046	0.047	0.000	0.050
	SOI-R desire	–0.007	0.029	–0.238	0.812	–0.064	0.050	0.000	0.056
	N of long-term relationships	–0.011	0.044	–0.244	0.808	–0.096	0.075	0.000	0.057
	N of short-term relationships	0.000	0.007	–0.040	0.968	–0.014	0.013	0.000	0.050
	Mate value–Long term	0.085	0.047	1.821	0.069	–0.007	0.176	0.006	0.443
	Mate value–Short term	0.042	0.051	0.835	0.404	–0.057	0.142	0.001	0.133
	BPA anger	–0.133	0.062	–2.164	0.031	–0.254	–0.012	0.009	0.579
	BPA physical aggression	0.153	0.079	1.941	0.053	–0.002	0.308	0.007	0.491
	BPA verbal aggression	0.237	0.070	3.379	0.001	0.099	0.375	0.021	0.921
	BPA hostility	–0.064	0.066	–0.960	0.337	–0.194	0.067	0.002	0.160
	IC attractiveness envy	–0.047	0.047	–0.986	0.324	–0.139	0.046	0.002	0.166
	IC superiority	0.065	0.045	1.435	0.152	–0.024	0.154	0.004	0.299
	IC status protection	0.010	0.044	0.237	0.813	–0.075	0.096	0.000	0.056
Circus arts	Intercept	0.249	0.377	0.660	0.510	–0.492	0.989	0.001	0.101
	Age	–0.008	0.006	–1.234	0.218	–0.020	0.004	0.003	0.234
	LH parenting effort	–0.112	0.042	–2.660	0.008	–0.195	–0.029	0.013	0.756
	LH mating effort	0.071	0.046	1.536	0.125	–0.020	0.161	0.004	0.335
	SOI-R behavior	–0.011	0.032	–0.358	0.721	–0.075	0.052	0.000	0.065
	SOI-R attitude	–0.056	0.022	–2.478	0.014	–0.100	–0.012	0.012	0.696
	SOI-R desire	0.008	0.027	0.279	0.780	–0.046	0.062	0.000	0.059
	N of long-term relationships	0.088	0.041	2.135	0.033	0.007	0.168	0.009	0.568
	N of short-term relationships	0.000	0.006	0.069	0.945	–0.012	0.013	0.000	0.051
	Mate value–Long term	0.065	0.044	1.487	0.138	–0.021	0.152	0.004	0.317
	Mate value–Short term	0.009	0.048	0.196	0.844	–0.085	0.103	0.000	0.054
	BPA anger	–0.008	0.058	–0.135	0.893	–0.122	0.106	0.000	0.052
	BPA physical aggression	0.039	0.075	0.521	0.603	–0.108	0.185	0.001	0.081
	BPA verbal aggression	–0.006	0.066	–0.087	0.930	–0.136	0.124	0.000	0.051
	BPA hostility	0.097	0.063	1.550	0.122	–0.026	0.220	0.005	0.340
	IC attractiveness envy	–0.016	0.045	–0.369	0.712	–0.104	0.071	0.000	0.066
	IC superiority	–0.020	0.043	–0.459	0.646	–0.104	0.064	0.000	0.074
	IC status protection	0.071	0.041	1.719	0.086	–0.010	0.152	0.006	0.404

*LH, life history; SOI-R, the sociosexual orientation inventory-revised; BPA, Buss-Perry Aggression questionnaire; IC, intrasexual competition.*

In men (*N* = 209), the model (Pillai’s Trace = 0.064, *F* = 2,571, *p* = 0.028, η_p_^2^ = 0.064, Observed power = 0.787) showed that Literary arts were predicted positively by Long-term mate value, IC-Superiority and verbal aggression, and negatively by hostility. Musical arts were predicted positively by IC-Status protection. Circus arts were positively predicted by Short-term mate value. There was no significant predictor for Visual arts. Sports were predicted positively by Short-term mate value, IC-Superiority and physical aggression, and negatively by anger. See Table 3.

**TABLE 3:** Parameter estimates of the general linear model (GLM) model indicating the effect of the independent variables on artisticality in men.

Dependent variable	Parameter	B	SE	*t*	*p*	95% CI		Partial eta^2^	Observed power
						Lower bound	Upper bound		
Literary arts	Intercept	0.091	0.636	0.144	0.886	–1.162	1.345	0.000	0.052
	Age	0.001	0.010	0.125	0.900	–0.019	0.022	0.000	0.052
	LH parenting effort	–0.022	0.067	–0.326	0.745	–0.155	0.111	0.001	0.062
	LH mating effort	–0.073	0.066	–1.109	0.269	–0.203	0.057	0.006	0.197
	SOI-R behavior	–0.002	0.051	–0.049	0.961	–0.102	0.097	0.000	0.050
	SOI-R attitude	–0.020	0.035	–0.570	0.569	–0.088	0.049	0.002	0.088
	SOI-R Desire	0.052	0.043	1.225	0.222	–0.032	0.136	0.008	0.230
	N of long-term relationships	0.036	0.049	0.733	0.465	–0.061	0.134	0.003	0.113
	N of short-term relationships	–0.004	0.009	–0.395	0.693	–0.022	0.014	0.001	0.068
	Mate value–Long term	0.189	0.064	2.952	0.004	0.063	0.315	0.044	0.836
	Mate value–Short term	0.049	0.071	0.693	0.489	–0.091	0.189	0.003	0.106
	BPA anger	0.100	0.096	1.035	0.302	–0.090	0.289	0.006	0.178
	BPA physical aggression	0.052	0.099	0.523	0.602	–0.143	0.246	0.001	0.081
	BPA verbal aggression	0.260	0.101	2.574	0.011	0.061	0.460	0.034	0.726
	BPA hostility	–0.334	0.104	–3.205	0.002	–0.539	–0.128	0.051	0.890
	IC attractiveness envy	0.007	0.084	0.083	0.934	–0.158	0.172	0.000	0.051
	IC superiority	0.157	0.070	2.241	0.026	0.019	0.296	0.026	0.606
	IC status protection	–0.062	0.067	–0.920	0.359	–0.194	0.071	0.004	0.150
Visual arts	Intercept	–1.995	0.653	–3.057	0.003	–3.282	–0.708	0.047	0.860
	Age	0.028	0.011	2.638	0.009	0.007	0.050	0.035	0.747
	LH parenting effort	0.069	0.069	0.997	0.320	–0.067	0.205	0.005	0.168
	LH mating effort	–0.044	0.068	–0.654	0.514	–0.178	0.089	0.002	0.100
	SOI-R behavior	–0.032	0.052	–0.610	0.543	–0.134	0.071	0.002	0.093
	SOI-R attitude	–0.046	0.036	–1.284	0.201	–0.116	0.025	0.009	0.248
	SOI-R desire	0.064	0.044	1.461	0.146	–0.022	0.150	0.011	0.307
	N of long-term relationships	0.008	0.051	0.159	0.874	–0.092	0.108	0.000	0.053
	N of short-term relationships	0.007	0.009	0.789	0.431	–0.011	0.026	0.003	0.123
	Mate value–Long term	0.047	0.066	0.708	0.480	–0.083	0.176	0.003	0.108
	Mate value–Short term	0.099	0.073	1.356	0.177	–0.045	0.242	0.010	0.271
	BPA anger	0.043	0.099	0.430	0.667	–0.152	0.237	0.001	0.071
	BPA physical aggression	–0.096	0.101	–0.951	0.343	–0.296	0.103	0.005	0.157
	BPA verbal aggression	0.172	0.104	1.658	0.099	–0.033	0.377	0.014	0.378
	BPA hostility	0.085	0.107	0.794	0.428	–0.126	0.296	0.003	0.124
	IC attractiveness envy	–0.062	0.086	–0.717	0.474	–0.231	0.108	0.003	0.110
	IC superiority	0.001	0.072	0.012	0.990	–0.141	0.143	0.000	0.050
	IC status protection	0.004	0.069	0.054	0.957	–0.132	0.140	0.000	0.050
Musical arts	Intercept	0.967	0.714	1.354	0.177	–0.441	2.374	0.010	0.271
	Age	–0.018	0.012	–1.499	0.135	–0.041	0.006	0.012	0.320
	LH parenting effort	0.073	0.076	0.961	0.338	–0.076	0.222	0.005	0.160
	LH mating effort	0.043	0.074	0.574	0.566	–0.104	0.189	0.002	0.088
	SOI-R behavior	0.091	0.057	1.611	0.109	–0.021	0.203	0.013	0.361
	SOI-R attitude	–0.027	0.039	–0.689	0.492	–0.104	0.050	0.002	0.105
	SOI-R desire	0.017	0.048	0.358	0.720	–0.077	0.112	0.001	0.065
	N of long-term relationships	0.002	0.055	0.035	0.972	–0.107	0.111	0.000	0.050
	N of short-term relationships	–0.001	0.010	–0.086	0.932	–0.021	0.019	0.000	0.051
	Mate value–Long term	0.081	0.072	1.130	0.260	–0.061	0.223	0.007	0.203
	Mate value–Short term	–0.106	0.080	–1.327	0.186	–0.263	0.051	0.009	0.262
	BPA anger	0.078	0.108	0.724	0.470	–0.135	0.291	0.003	0.111
	BPA physical aggression	–0.188	0.111	–1.698	0.091	–0.407	0.030	0.015	0.394
	BPA verbal aggression	–0.203	0.114	–1.791	0.075	–0.427	0.021	0.017	0.429
	BPA hostility	–0.208	0.117	–1.780	0.077	–0.439	0.023	0.016	0.425
	IC attractiveness envy	0.150	0.094	1.600	0.111	–0.035	0.335	0.013	0.357
	IC superiority	0.044	0.079	0.555	0.579	–0.112	0.199	0.002	0.086
	IC status protection	0.155	0.075	2.058	0.041	0.006	0.304	0.022	0.535
Sports	Intercept	0.607	0.681	0.892	0.374	–0.736	1.950	0.004	0.144
	Age	–0.024	0.011	–2.146	0.033	–0.046	–0.002	0.024	0.570
	LH parenting effort	0.099	0.072	1.376	0.170	–0.043	0.241	0.010	0.278
	LH mating effort	0.002	0.071	0.033	0.974	–0.137	0.142	0.000	0.050
	SOI-R behavior	0.034	0.054	0.633	0.527	–0.072	0.141	0.002	0.097
	SOI-R attitude	0.052	0.037	1.392	0.166	–0.022	0.125	0.010	0.283
	SOI-R desire	–0.084	0.046	–1.850	0.066	–0.174	0.006	0.018	0.453
	N of long-term relationships	0.019	0.053	0.366	0.715	–0.085	0.124	0.001	0.065
	N of short-term relationships	7.382E-5	0.010	0.008	0.994	–0.019	0.019	0.000	0.050
	Mate value–Long term	0.071	0.069	1.042	0.299	–0.064	0.207	0.006	0.179
	Mate value–Short term	0.279	0.076	3.673	0.000	0.129	0.428	0.066	0.955
	BPA anger	–0.263	0.103	–2.555	0.011	–0.467	–0.060	0.033	0.720
	BPA physical aggression	0.238	0.106	2.256	0.025	0.030	0.447	0.026	0.612
	BPA verbal aggression	–0.053	0.108	–0.486	0.628	–0.266	0.161	0.001	0.077
	BPA hostility	0.016	0.112	0.142	0.888	–0.204	0.236	0.000	0.052
	IC attractiveness envy	0.007	0.089	0.082	0.935	–0.169	0.184	0.000	0.051
	IC superiority	0.235	0.075	3.122	0.002	0.087	0.383	0.049	0.874
	IC status protection	0.046	0.072	0.639	0.524	–0.096	0.188	0.002	0.097
Circus arts	Intercept	0.412	0.792	0.521	0.603	–1.150	1.974	0.001	0.081
	Age	–0.002	0.013	–0.143	0.886	–0.028	0.024	0.000	0.052
	LH parenting effort	0.052	0.084	0.616	0.539	–0.114	0.217	0.002	0.094
	LH mating effort	–0.071	0.082	–0.861	0.390	–0.233	0.091	0.004	0.137
	SOI-R behavior	–0.059	0.063	–0.931	0.353	–0.183	0.066	0.005	0.153
	SOI-R attitude	–0.012	0.043	–0.270	0.787	–0.097	0.074	0.000	0.058
	SOI-R desire	0.034	0.053	0.637	0.525	–0.071	0.139	0.002	0.097
	N of long-term relationships	–0.054	0.062	–0.879	0.380	–0.175	0.067	0.004	0.141
	N of short-term relationships	0.008	0.011	0.707	0.481	–0.014	0.031	0.003	0.108
	Mate value–Long term	0.108	0.080	1.358	0.176	–0.049	0.266	0.010	0.272
	Mate value–Short term	0.188	0.088	2.135	0.034	0.014	0.363	0.023	0.565
	BPA anger	–0.011	0.120	–0.092	0.927	–0.248	0.225	0.000	0.051
	BPA physical aggression	0.113	0.123	0.919	0.359	–0.129	0.355	0.004	0.150
	BPA verbal aggression	–0.119	0.126	–0.942	0.347	–0.367	0.130	0.005	0.155
	BPA hostility	0.129	0.130	0.994	0.322	–0.127	0.385	0.005	0.167
	IC attractiveness envy	–0.020	0.104	–0.194	0.846	–0.225	0.185	0.000	0.054
	IC superiority	0.090	0.088	1.025	0.306	–0.083	0.262	0.005	0.175
	IC status protection	0.052	0.084	0.627	0.532	–0.113	0.218	0.002	0.096

*LH, life history; SOI-R, the sociosexual orientation inventory-revised; BPA, Buss-Perry Aggression questionnaire; IC, intrasexual competition.*

## Discussion

We investigated the relative importance of intersexual and intrasexual selection as possible evolutionary mechanisms maintaining human artistic and athletic propensities. We tested whether individual differences in artistic and athletic propensities covary with traits related to inter- or intrasexual selection in both sexes in Brazilian and Czech samples. We found that although inter- and intrasexual selections are present to a certain degree in both sexes, each sexual selection component has different relative importance in each sex. This finding is aligned with the “beauty and the beast” model (Puts, 2010), in which intrasexual competition is argued to be more relevant in men and intersexual selection more relevant in women throughout human evolution. Moreover, in line with the empirical review pointing to a slight superiority of women in the aesthetic and artistic domains (Varella et al., 2017), we found higher overall effect sizes and more significant relationships between the artistic propensities and the sexual selection processes in women than in men. In women, literary and musical arts were related to both elevated inter- and intrasexual selection proxies; visual and circus arts were related to elevated intersexual selection proxies; while sports were only related to intrasexual selection proxies. In men, literary arts and sports were related to both elevated inter- and intrasexual selections proxies; musical arts were related to a single elevated intrasexual proxy, and circus arts were related to a single elevated intersexual proxy. Thus, in accordance to the Mutual Mate Choice model (Stewart-Williams and Thomas, 2013), as for other physical and vocal traits (Puts, 2010; Puts et al., 2016; Saxton et al., 2016; Kordsmeyer et al., 2018), both components of sexual selection might have been among the influential selective pressures acting upon the ancestral evolution of human artisticality (De Block and Dewitte, 2007; Varella et al., 2017) and athleticism (Lombardo, 2012; Apostolou, 2015; Deaner et al., 2016).

In women, artistic tendencies were associated with nine proxies of intersexual selection (five positive and four negative) and seven proxies of intrasexual competition (four negative and three positive). For literary arts, despite the negative association with SOI-R Attitude, we found a positive association between short-term mate value and sociosexual desire, conceptually, replicating the relationship between humor and casual sex (Greengross and Miller, 2011), and that poets have more sexual partners (Nettle and Clegg, 2006). Moreover, literary arts were also positively related to the superiority dimension of Intrasexual Competition (i.e., IC-superiority) indicating an ornamental competition facet in asserting superiority over rivals (Varella et al., 2017). In visual arts, we found a positive association with parenting effort and negative associations with IC-Attractiveness envy and anger which indicates a clear intersexual selection pattern focused on maintaining a long-term bond, collaboration, and reducing conflict. In musical arts, we found positive associations with short-term mate value and IC-Attractiveness envy and negative associations with SOI-R Attitude and hostility, indicating a specific combination of intra- and intersexual selection. This pattern supports the finding of explicit intrasexual competitive strategies in the song lyrics of female pop musicians (Fisher and Candea, 2012). Importantly, our musical arts factor was composed of singing, playing musical instruments, and dancing. Dancing was more distant from the two previous items, and future studies might focus on specific differences between auditory and body movement music modalities. For circus arts, we showed that the tendencies to aesthetically enhance bodily movements are positively related to the number of long-term partners and negatively to parenting effort and SOI-R Attitude, indicating a higher serial monogamy pattern, although it leads to higher reproductive success in men than in women (Jokela et al., 2010).

In men, seven proxies of intrasexual competition (five positive and two negative), and three proxies of intersexual selection were (positively) associated with artistic tendencies. In literary arts, we found a positive association with long-term mate value which disagree but mirrors previous findings in which humor and poetry relates to short-term mating (Nettle and Clegg, 2006; Greengross and Miller, 2011). Poetry, humor, and creative writing might have slightly different effects, and some of them might be more related to short-term, while others to long-term, sexual strategies. Moreover, literary arts were positively related to the IC-superiority and verbal aggression (although negatively related to hostility), which are possibly useful tendencies in gaining prestige against competitors within this highly language-mediated modality. The general pattern of associations in literary arts was the only similarity for both sexes. In musical arts, we found a positive connection with the IC-protection of status, which is in accordance with findings that the hierarchy of musical ability within the orcherstra is associated to a more masculine digit lenght ratio (Sluming and Manning, 2000), male physical size positively predicts speech and singing attractiveness (Valentova et al., 2019), male physical strength predicts dance attractiveness (Hugill et al., 2009; Weege et al., 2015), and “good” male dances are rated as more masculine (Weege et al., 2012). Although we did not capture the intersexual component, our results shed some light on the reason why cues and perceptions of masculinity are aligned with attractiveness ratings. Women might be detecting indications of men’s ability to win intrasexual competition and consequently viewing these men as attractive (Weege et al., 2012, 2015). We did not replicate the positive relationship with sociosexuality (Valentova et al., 2019), although in that study that finding was restricted to higher-pitched male singing and not to the entire musical arts factor. In visual arts, we had no significant predictor, which does not corroborate the finding that successful male visual artists had more sexual partners and a long-term oriented sexual strategy (Clegg et al., 2011). Finally, we showed that the tendencies to aesthetically enhance bodily movements (circus arts) are positively related to mate value for short-term relationships, which supports the proposal that male bodily strength is the best predictor of mating and reproductive success (Lidborg et al., 2022).

Artisticality is not the only route to compete for, create and/or maintain sexual relationships potentially leading to differential reproductive success. Athleticism was related to intrasexual competition in both sexes (i.e., less anger in both sexes and more verbal aggression in women, and more physical aggression and intrasexual competition superiority in men) corroborating Graves (2010); Lombardo (2012), and Longman et al. (2020), and expanding their logic to include women. Moreover, athleticism was associated to high short-term mate value in men, corroborating Zahavi and Zahavi (1999) and Miller (2000). Although we did not include all dimensions of physical activities, such as exercises or body practice which might have different motivations than sports (Hsu and Valentova, 2020), we corroborate studies proposing that sexual selection could have influenced athleticism and sports activities (De Block and Dewitte, 2009; Graves, 2010; Lombardo, 2012; Apostolou, 2015; Deaner et al., 2016; Longman et al., 2020).

We relied on a convenience sample of heterosexual individuals in our correlational study, and thus we cannot propose any unidirectional causal links. However, our samples were from two disparate cultures which improves generalizability. Importantly, the biggest difference between our and some previous studies is that we sampled the “common” population, not professional artists or athletes which may explain the relatively weak or no associations in our study. However, even weak associations among a general population show the potential of art-related behavior in sexual selection. We did not control for public versus private artistic display, or for success and professional arts, which are factors important in sexual selection, and should be the focus of future studies. Our study does not allow for an in-depth examination within each artistic/sport factor. Future studies should explore the specific intra/intersexual correlates for each leisure activity because they might differ in the level of skill or aggressiveness involved which could translate into being used more as an ornament or an armament. For example, boxing is quite different from golf, and likewise, the status of lead guitarists is dissimilar from those playing a keyboard in the background.

This cross-cultural study indicated that sports and each artistic modality exhibit their own sexual selection pattern and each could possibly yield different individual qualities (cf. Sluming and Manning, 2000; Candolin, 2003; Valentova et al., 2017; Pereira et al., 2019). In general, artistic propensity is influenced by both intra- and intersexual selection for both sexes, however, we found more intrasexual competition proxies in men and slightly more intersexual proxies in women. Further, there were more relationships between artistic propensities and sexual selection in women, and between athletic propensities and sexual selection in men. Thus, artistic tendencies in humans can serve the dual function of attracting/maintaining short- or long-term partners, and as tactics for competing with rivals. Athletic tendencies were related to intrasexual competition proxies in both sexes and intersexual proxy only in men, indicating that they might serve a dual function in men while serving more as an armament in women. Therefore, we expanded the scope of sexual section processes to show their relative and simultaneous influences. Future studies should explore different sexual selection processes and also survival values to test their relative importance across a higher cultural diversity.

## Data Availability Statement

The datasets presented in this study can be found in online repositories. The names of the repository/repositories and accession number(s) can be found below: Open Science Framework https://osf.io/59cjb/.

## Ethics Statement

Ethical review and approval was not required for the study on human participants in accordance with the local legislation and institutional requirements. The patients/participants provided their written informed consent to participate in this study.

## Author Contributions

MV, MF, and JV conceived and designed the study. MV searched the literature and drafted the manuscript. MV, JV, ZŠ, and KB gathered the data. JV organized and analyzed the data and wrote the results. All authors wrote, revised, and approved the final manuscript.

## Conflict of Interest

The authors declare that the research was conducted in the absence of any commercial or financial relationships that could be construed as a potential conflict of interest.

## Publisher’s Note

All claims expressed in this article are solely those of the authors and do not necessarily represent those of their affiliated organizations, or those of the publisher, the editors and the reviewers. Any product that may be evaluated in this article, or claim that may be made by its manufacturer, is not guaranteed or endorsed by the publisher.

## References

[B1] Andersson M. B. (1994). *Sexual Selection.* Princeton, NJ: Princeton University Press.

[B2] Andrews P. W. Gangestad S. W. Matthews D. (2002). Adaptationism–how to carry out an exaptationist program. *Behav. Brain Sci.* 25 489–504. 10.1017/s0140525x02000092 12879701

[B3] Apostolou M. (2015). The athlete and the spectator inside the man: a cross-cultural investigation of the evolutionary origins of athletic behavior. *Cross Cult. Res.* 49 151–173. 10.1177/1069397114536516

[B4] Beaussart M. L. Kaufman S. B. Kaufman J. C. (2012). Creative activity, personality, mental illness, and short-term mating success. *J. Creat. Behav.* 46 151–167. 10.1002/jocb.11

[B5] Bongard S. Schulz I. Studenroth K. U. Frankenberg E. (2019). Attractiveness ratings for musicians and non-musicians: an evolutionary-psychology perspective. *Front. Psychol.* 10:2627. 10.3389/fpsyg.2019.02627 31849756 PMC6895061

[B6] Bressler E. R. Balshine S. (2006). The influence of humor on desirability. *Evol. Hum. Behav.* 27 29–39. 10.1016/j.evolhumbehav.2005.06.002

[B7] Bressler E. R. Martin R. A. Balshine S. (2006). Production and appreciation of humor as sexually selected traits. *Evol. Hum. Behav.* 27 121–130. 10.1016/j.evolhumbehav.2005.09.001

[B8] Buss D. M. Abbott M. Angleitner A. Asherian A. Biaggio A. Blanco-Villasenor A. (1990). International preferences in selecting mates: a study of 37 cultures. *J. Cross Cult. Psychol.* 21 5–47. 10.1177/0022022190211001

[B9] Buss D. M. Barnes M. (1986). Preferences in human mate selection. *J. Pers. Soc. Psychol.* 50 559–570. 10.1037/0022-3514.50.3.559

[B10] Buunk A. P. Fisher M. (2009). Individual differences in intrasexual competition. *J. Evol. Psychol.* 7 37–48. 10.1556/JEP.7.2009.1.5

[B11] Candolin U. (2003). The use of multiple cues in mate choice. *Biol. Rev.* 78 575–595. 10.1017/S1464793103006158 14700392

[B12] Carson S. H. Peterson J. B. Higgins D. M. (2005). Reliability, validity, and factor structure of the creative achievement questionnaire. *Creat. Res. J.* 17 37–50. 10.1207/s15326934crj1701_4

[B13] Chang A. Kragness H. E. Tsou W. Bosnyak D. J. Thiede A. Trainor L. J. (2021). Body sway predicts romantic interest in speed dating. *Soc. Cogn. Affect. Neurosci.* 16 185–192. 10.1093/scan/nsaa093 32685965 PMC7812630

[B14] Charlton B. D. (2014). Menstrual cycle phase alters women’s sexual preferences for composers of more complex music. *Proc. R. Soc. B: Biol. Sci.* 281:20140403. 10.1098/rspb.2014.0403 24759864 PMC4043099

[B15] ChenB. B.ChangL. (2015). Creativity and aggression as ornament and armament: intersexual and intrasexual selection on men’s mating behaviors. *Evol. Psychol.* 13:147470491501300118. 10.1177/14747049150130011825812074

[B16] Clegg H. Nettle D. Miell D. (2011). Status and mating success amongst visual artists. *Front. Psychol.* 2:310. 10.3389/fpsyg.2011.00310 22059085 PMC3204576

[B17] Darwin C. (1871). *The Descent of Man, and Selection in Relation to Sex.* London: John Murray.

[B18] Davies S. (2012). *The Artful Species: Aesthetics, Art, and Evolution.* Oxford: Oxford University Press.

[B19] De Block A. Dewitte S. (2007). Mating games: cultural evolution and sexual selection. *Biol. Philos.* 22 475–491. 10.1007/s10539-006-9041-y

[B20] De Block A. Dewitte S. (2009). Darwinism and the cultural evolution of sports. *Perspect. Biol. Med.* 52 1–16. 10.1353/pbm.0.0063 19168940

[B21] De Tiège A. Verpooten J. Braeckman J. (2021). From animal signals to art: manipulative animal signaling and the evolutionary foundations of aesthetic behavior and art production. *Q. Rev. Biol.* 96 1–27. 10.1086/713210

[B22] Deaner R. O. Balish S. M. Lombardo M. P. (2016). Sex differences in sports interest and motivation: an evolutionary perspective. *Evol. Behav. Sci.* 10 73–97. 10.1037/ebs0000049

[B23] Dissanayake E. (2008). “The arts after darwin: does art have an origin and adaptive function?,” in *World Art Studies: Exploring Concepts and Approaches*, eds Zijlemans K. van Damme W. (Amsterdam: Valiz), 241–263. 10.1007/s13187-012-0411-7

[B24] Dutton D. (2009). *The Art Instinct.* Oxford: Oxford University Press.

[B25] Eibl-Eibesfeldt I. (1989). *Human Ethology.* New York: Aldine de Gruyter.

[B26] Fernandez A. M. Muñoz-Reyes J. A. Dufey M. (2014). BMI, age, mate value, and intrasexual competition in Chilean women. *Curr. Psychol.* 33 435–450. 10.1007/s12144-014-9221-x

[B27] Fink B. Hugill N. Lange B. P. (2012). Women’s body movements are a potential cue to ovulation. *Pers. Individ. Differ.* 53 759–763. 10.1016/j.paid.2012.06.005

[B28] Fisher M. L. Candea C. (2012). You ain’t woman enough to take my man: female intrasexual competition as portrayed in songs. *J. Soc. Evol. Cult. Psychol.* 6:480. 10.1037/h0099238

[B29] Fukui H. (2001). Music and testosterone: a new hypothesis for the origin and function of music. *Ann. N.Y. Acad. Sci.* 930 448–451. 10.1111/j.1749-6632.2001.tb05767.x 11458865

[B30] Galasinska K. Szymkow A. (2021). The more fertile, the more creative: changes in women’s creative potential across the ovulatory cycle. *J. Environ. Res. Public Health* 18:5390. 10.3390/ijerph18105390 34070114 PMC8158362

[B31] Gouveia V. V. Chaves C. M. C. M. Peregrino R. R. Branco A. O. C. Gonçalves M. P. (2008). Medindo a agressão: O questionário de buss-perry. *Arq. Bras. Psicol.* 60 92–103.

[B32] Graves B. M. (2010). Ritualized combat as an indicator of intrasexual selection effects on male life history evolution. *Am. J. Hum. Biol.* 22 45–49. 10.1002/ajhb.20933 19384865

[B33] Greengross G. Miller G. (2011). Humor ability reveals intelligence, predicts mating success, and is higher in males. *Intelligence* 39 188–192. 10.1016/j.intell.2011.03.006

[B34] Griskevicius V. Cialdini R. B. Kenrick D. T. (2006). Peacocks, picasso, and parental investment: the effects of romantic motives on creativity. *J. Pers. Soc. Psychol.* 91:63. 10.1037/0022-3514.91.1.63 16834480

[B35] Harrison M. A. Hughes S. M. (2017). Sex drugs and rock and roll: evidence supporting the storied trilogy. *Hum. Ethol. Bull.* 32 63–84. 10.22330/heh/323/63-84

[B36] Haselton M. G. Miller G. F. (2006). Women’s fertility across the cycle increases the short-term attractiveness of creative intelligence. *Hum. Nat.* 17 50–73. 10.1007/s12110-006-1020-0 26181345

[B37] Hodges-Simeon C. R. Gaulin S. J. Puts D. A. (2011). Voice correlates of mating success in men: examining “contests” versus “mate choice” modes of sexual selection. *Arch. Sex. Behav.* 40 551–557. 10.1007/s10508-010-9625-0 20369377

[B38] Hodgson D. Verpooten J. (2015). The evolutionary significance of the arts: exploring the by-product hypothesis in the context of ritual, precursors, and cultural evolution. *Biol. Theory* 10 73–85.

[B39] Hogh-Olesen H. (2018). *The Aesthetic Animal.* Oxford: Oxford University Press.

[B40] HoneL. S.HurwitzW.LiebermanD. (2015). Sex differences in preferences for humor: a replication, modification, and extension. *Evol. Psychol.* 13:147470491501300110. 10.1177/14747049150130011025670631

[B41] Hsu R. M. C. S. Valentova J. V. (2020). Motivation for different physical activities: a comparison among sports, exercises and body/movement practices. *Psicol. USP* 31:153. 10.1590/0103-6564e190153

[B42] Hsu R. M. C. S. Cardoso F. L. Varella M. A. C. Pires E. M. Valentova J. V. (2022). Comparing different typologies of physical activities with a focus on motivation. *Front. Psychol.* 2022:2194. 10.3389/fpsyg.2022.790490 35645925 PMC9137393

[B43] Hugill N. Fink B. Neave N. Seydel H. (2009). Men’s physical strength is associated with women’s perceptions of their dancing ability. *Pers. Individ. Differ.* 47 527–530. 10.1016/j.paid.2009.04.009

[B44] Jokela M. Rotkirch A. Rickard I. J. Pettay J. Lummaa V. (2010). Serial monogamy increases reproductive success in men but not in women. *Behav. Ecol.* 21 906–912. 10.1093/beheco/arq078

[B45] Kalinowski K. Kozłowska A. Malesza M. Danel D. P. (2021). Evolutionary origins of music. classical and recent hypotheses. *Anthropol. Rev.* 84 213–231. 10.2478/anre-2021-0011

[B46] Kamble S. Shackelford T. K. Pham M. Buss D. M. (2014). Indian mate preferences: continuity, sex differences, and cultural change across a quarter of a century. *Pers. Individ. Differ.* 70 150–155. 10.1016/j.paid.2014.06.024

[B47] Karamihalev S. (2013). Why creativity is sexy: a review of the evidence of sexual selection for creative abilities in humans. *J. Eur. Psychol. Stud.* 4 78–86. 10.5334/jeps.bb

[B48] Kaufman S. B. Kozbelt A. Silvia P. Kaufman J. C. Ramesh S. Feist G. J. (2016). Who finds bill gates sexy? Creative mate preferences as a function of cognitive ability, personality, and creative achievement. *J. Creat. Behav.* 50 294–307. 10.1002/jocb.78

[B49] Kordsmeyer T. L. Hunt J. Puts D. A. Ostner J. Penke L. (2018). The relative importance of intra-and intersexual selection on human male sexually dimorphic traits. *Evol. Hum. Behav.* 39 424–436. 10.1016/j.evolhumbehav.2018.03.008

[B50] Kruger D. J. (2017). Brief self-report scales assessing life history dimensions of mating and parenting effort. *Evol. Psychol.* 15:1474704916673840. 10.1177/1474704916673840 28152624 PMC10481099

[B51] Lebuda I. Sorokowski P. Groyecka-Bernard A. Marczak M. Gajda A. Jankowska D. M. (2021). Creativity, mating, and reproductive successes outside the WEIRD world. *Creat. Res. J.* 33 255–263. 10.1080/10400419.2020.1870816

[B52] Li N. P. Bailey J. M. Kenrick D. T. Linsenmeier J. A. (2002). The necessities and luxuries of mate preferences: testing the tradeoffs. *J. Pers. Soc. Psychol.* 82 947–955. 10.1037/0022-3514.82.6.947 12051582

[B53] Lidborg L. H. Cross C. P. Boothroyd L. G. (2022). A meta-analysis of the association between male dimorphism and fitness outcomes in humans. *Elife* 11:e65031. 10.7554/eLife.65031 35179485 PMC9106334

[B54] LombardoM. P. (2012). On the evolution of sport. *Evol. Psychol.* 10 1–28. 10.1177/14747049120100010122833842

[B55] Longman D. P. Wells J. C. Stock J. T. (2020). Human athletic paleobiology: using sport as a model to investigate human evolutionary adaptation. *Am. J. Phys. Anthropol.* 171 42–59. 10.1002/ajpa.23992 31957878 PMC7217212

[B56] Low B. S. (1979). “Sexual selection and human ornamentation,” in *Evolutionary Biology And Human Social Behavior*, eds Chagnon N. A. Irons W. (Boston: Duxbury Press), 462–487.

[B57] Mafra A. L. Varella M. A. C. Defelipe R. P. Anchieta N. M. de Almeida C. A. G. Valentova J. V. (2020). Makeup usage in women as a tactic to attract mates and compete with rivals. *Pers. Individ. Differ.* 163:110042. 10.1016/j.paid.2020.110042

[B58] Marin M. M. Schober R. Gingras B. Leder H. (2017). Misattribution of musical arousal increases sexual attraction towards opposite-sex faces in females. *PLoS One* 12:e0183531. 10.1371/journal.pone.0183531 28892486 PMC5593195

[B59] Martindale C. Locher P. Petrov V. M. Berleant A. (2007). *Evolutionary and Neurocognitive Approaches to Aesthetics, Creativity and the Arts.* New York: Taylor and Francis.

[B60] May J. L. Hamilton P. A. (1980). Effects of musically evoked affect on women’s interpersonal attraction toward and perceptual judgments of physical attractiveness of men. *Motiv. Emot.* 4 217–228. 10.1007/BF00995420

[B61] Menninghaus W. (2019). *Aesthetics After Darwin: The Multiple Origins and Functions of Art.* Boston: Academic Studies Press.

[B62] Miller G. (2000). *The Mating Mind.* New York, NY: Anchor Books.

[B63] Miller G. Tybur J. M. Jordan B. D. (2007). Ovulatory cycle effects on tip earnings by lap dancers: economic evidence for human estrus? *Evol. Hum. Behav.* 28 375–381. 10.1016/j.evolhumbehav.2007.06.002

[B64] Moraes Y. L. Valentova J. V. Varella M. A. C. (2021). The evolution of playfulness, and selection in relation to sex. *Front. Psychol.* 2022:925842. 10.3389/fpsyg.2022.925842 35756316 PMC9226980

[B65] Mosing M. A. Verweij K. J. Madison G. Pedersen N. L. Zietsch B. P. Ullén F. (2015). Did sexual selection shape human music? Testing predictions from the sexual selection hypothesis of music evolution using a large genetically informative sample of over 10,000 twins. *Evol. Hum. Behav.* 36 359–366. 10.1016/j.evolhumbehav.2015.02.004

[B66] Nettle D. Clegg H. (2006). Schizotypy, creativity and mating success in humans. *Proc. R. Soc. B.* 273 611–615. 10.1098/rspb.2005.3349 16537133 PMC1560060

[B67] Penke L. Asendorpf J. B. (2008). Beyond global sociosexual orientations: a more differentiated look at sociosexuality and its effects on courtship and romantic relationships. *J. Pers. Soc. Psychol.* 95:1113. 10.1037/0022-3514.95.5.1113 18954197

[B68] Pereira K. J. Varella M. A. C. Kleisner K. Pavlovič O. Valentova J. V. (2019). Positive association between facial and vocal femininity/masculinity in women but not in men. *Behav. Process.* 164 25–29. 10.1016/j.beproc.2019.04.010 31002841

[B69] Pinker S. (2004). *The Blank Slate: The Modern Denial of Human Nature.* New York, NY: Viking.

[B70] Prokosch M. D. Coss R. G. Scheib J. E. Blozis S. A. (2009). Intelligence and mate choice: intelligent men are always appealing. *Evol. Hum. Behav.* 30 11–20. 10.1016/j.evolhumbehav.2008.07.004

[B71] Puts D. A. (2010). Beauty and the beast: mechanisms of sexual selection in humans. *Evol. Hum. Behav.* 31 157–175. 10.1016/j.evolhumbehav.2010.02.005

[B72] Puts D. A. Barndt J. L. Welling L. L. Dawood K. Burriss R. P. (2011). Intrasexual competition among women: vocal femininity affects perceptions of attractiveness and flirtatiousness. *Pers. Individ. Differ.* 50 111–115. 10.1016/j.paid.2010.09.011

[B73] Puts D. A. Hill A. K. Bailey D. H. Walker R. S. Rendall D. Wheatley J. R. (2016). Sexual selection on male vocal fundamental frequency in humans and other anthropoids. *Proc. R. Soc. B Biol. Sci.* 283:20152830. 10.1098/rspb.2015.2830 27122553 PMC4855375

[B74] Richards R. A. (2019). *The Biology of Art.* Cambridge: Cambridge University Press.

[B75] Röder S. Carbon C.-C. Shackelford T. K. Pisanski K. Weege B. Fink B. (2016). Men’s visual attention and perceptions of women’s dance movements. *Pers. Individ. Differ.* 101 1–3. 10.1016/j.paid.2016.05.025

[B76] Saxton T. K. Mackey L. L. McCarty K. Neave N. (2016). A lover or a fighter? Opposing sexual selection pressures on men’s vocal pitch and facial hair. *Behav. Ecol.* 27 512–519. 10.1093/beheco/arv178 27004013 PMC4797380

[B77] Schmitt D. P. Pilcher J. J. (2004). Evaluating evidence of psychological adaptation: how do we know one when we see one? *Psychol. Sci.* 15 643–649. 10.1111/j.0956-7976.2004.00734.x 15447633

[B78] Semenyna S. W. Gómez Jiménez F. R. VanderLaan D. P. Vasey P. L. (2020). Inter-sexual mate competition in three cultures. *PLoS One* 15:e0236549. 10.1371/journal.pone.0236549 32726326 PMC7390379

[B79] Sluming V. A. Manning J. T. (2000). Second to fourth digit ratio in elite musicians: evidence for musical ability as an honest signal of male fitness. *Evol. Hum. Behav.* 21 1–9. 10.1016/S1090-5138(99)00026-4

[B80] Souza A. L. Conroy-Beam D. Buss D. M. (2016). Mate preferences in Brazil: evolved desires and cultural evolution over three decades. *Pers. Individ. Differ.* 95 45–49. 10.1016/j.paid.2016.01.053

[B81] Souza R. (2004). Is art an adaptation? Prospects for an evolutionary perspective on aesthetic emotions. *J. Aesthet. Art Crit.* 62 109–118. 10.1111/j.1540-594X.2004.00144.x

[B82] Stewart-Williams S. Thomas A. G. (2013). The ape that thought it was a peacock: does evolutionary psychology exaggerate human sex differences? *Psychol. Inq.* 24 137–168. 10.1080/1047840X.2013.804899

[B83] Sütterlin C. Schiefenhövel W. Lehmann C. Forster J. Apfelauer G. (2014). “Art as behaviour – an ethological approach to visual and verbal art, music and architecture,” in *Hanse Studies*, eds Sütterlin C. Schiefenhövel W. Lehmann C. Forster J. Apfelauer G. (Oldenburg: BIS-Verlag der Carl von Ossietzky Universität Oldenburg).

[B84] Thomas A. G. Jonason P. K. Blackburn J. D. Kennair L. E. O. Lowe R. Malouff J. (2020). Mate preference priorities in the east and west: a cross-cultural test of the mate preference priority model. *J. Pers.* 88 606–620. 10.1111/jopy.12514 31494937

[B85] Tifferet S. Gaziel O. Baram Y. (2012). Guitar increases male facebook attractiveness: preliminary support for the sexual selection theory of music. *Lett. Evol. Behav. Sci.* 3 4–6. 10.5178/lebs.2012.18

[B86] Valentova J. V. Junior F. P. M. Štìrbová Z. Varella M. A. C. Fisher M. L. (2020). The association between dark triad traits and sociosexuality with mating and parenting efforts: a cross-cultural study. *Pers. Individ. Differ.* 154:109613. 10.1016/j.paid.2019.109613

[B87] Valentova J. V. Tureček P. Varella M. A. C. Šebesta P. Mendes F. D. C. Pereira K. J. (2019). Vocal parameters of speech and singing covary and are related to vocal attractiveness, body measures, and sociosexuality: a cross-cultural study. *Front. Psychol.* 10:2029. 10.3389/fpsyg.2019.02029 31695631 PMC6817625

[B88] Valentova J. V. Varella M. A. C. Havlíček J. Kleisner K. (2017). Positive association between vocal and facial attractiveness in women but not in men: a cross-cultural study. *Behav. Process.* 135 95–100. 10.1016/j.beproc.2016.12.005 27986472

[B89] Varella M. A. C. (2021). Evolved features of artistic motivation: analyzing a brazilian database spanning three decades. *Front. Psychol.* 12:769915. 10.3389/fpsyg.2021.769915 34992565 PMC8724029

[B90] Varella M. A. C. Ferreira J. H. B. P. Cosentino L. A. M. Ottoni E. B. Bussab V. S. R. (2010). Sex differences in aspects of musicality in a brazilian sample: adaptive hypotheses. *Cogn. Music. Arts* 4 10–16.

[B91] Varella M. A. C. Santos I. B. C. Ferreira J. H. B. P. Bussab V. S. R. (2013). Misunderstandings in applying evolution to human mind and behavior and its causes: a systematic review. *EvoS J.* 5 81–107.

[B92] Varella M. A. C. Souza A. A. L. Ferreira J. H. B. P. (2011). Evolutionary aesthetics and sexual selection in the evolution of rock art aesthetics. *Rock Art Res.* 28 153–163.

[B93] Varella M. A. C. Souza A. A. L. Ferreira J. H. B. P. (2012). Considering both proximal and distal explanations for (rock) art production and appreciation as fruitful. *Rock Art Res.* 29 227–229.

[B94] Varella M. A. C. Valentova J. V. Fernández A. M. (2017). “Evolution of artistic and aesthetic propensities through female competitive ornamentation,” in *The Oxford Handbook of Female Competition*, ed. Fisher M. L. (New York, NY: Oxford University Press), 757–783. 10.1093/oxfordhb/9780199376377.013.46

[B95] Watkins C. D. (2017). Creating beauty: creativity compensates for low physical attractiveness when individuals assess the attractiveness of social and romantic partners. *R. Soc. Open Sci.* 4:160955. 10.1098/rsos.160955 28484614 PMC5414251

[B96] Weege B. Lange B. P. Fink B. (2012). Women’s visual attention to variation in men’s dance quality. *Pers. Individ. Differ.* 53 236–240. 10.1016/j.paid.2012.03.011

[B97] Weege B. Pham M. N. Shackelford T. K. Fink B. (2015). Physical strength and dance attractiveness: further evidence for an association in men, but not in women. *Am. J. Hum. Biol.* 27 728–730. 10.1002/ajhb.22703 25754668

[B98] Winegard B. Winegard B. Geary D. C. (2018). The status competition model of cultural production. *Evol. Psychol. Sci.* 4 351–371. 10.1007/s40806-018-0147-7

[B99] Zahavi A. Zahavi A. (1999). *The Handicap Principle: A Missing Piece of Darwin’s Puzzle.* Oxford: Oxford University Press.

